# Potential Acupoint Prescriptions and Outcome Reporting for Acupuncture in Atopic Eczema: A Scoping Review

**DOI:** 10.1155/2021/9994824

**Published:** 2021-06-26

**Authors:** Zhiwen Zeng, Man Li, Yunjie Zeng, Jialing Zhang, Yingjie Zhao, Yuanxun Lin, Ruijin Qiu, Dong-Shu Zhang, Hong-Cai Shang

**Affiliations:** ^1^Nanfang Hospital, Southern Medical University, Guangzhou, Guangdong, China; ^2^School of Traditional Chinese Medicine, Southern Medical University, Guangzhou, Guangdong, China; ^3^Dongzhimen Hospital of Beijing University of Chinese Medicine, Beijing, China

## Abstract

**Background:**

Acupuncture is considered a complementary therapy for atopic eczema. The aim of this scoping review is to identify, examine, and summarize the potential acupoint prescriptions and outcome reporting regarding the clinical trials of acupuncture for eczema.

**Methods:**

We searched different databases from inception to September 30, 2020. The data were screened and extracted to identify the potential acupuncture prescription and examine the variation in outcome reporting, outcome measurement instruments (OMIs), and measurement time points in clinical trials of acupuncture.

**Results:**

A total of 116 clinical studies were included. The acupoint combination of LI11 and SP10 was used frequently. The core acupoint association networks were acupoints LI11, SP10, ST36, SP6, and LI4. For clinical trials of acupuncture, a total of 6 outcome distinct domains were identified in the 32 outcome measurements. The most frequently reported outcome was the eczema area, which was reported 97 times (83.6%, 97/116). Immune system outcomes were assessed in 15 outcome measurements, which totally reported 37 times. Adverse events were reported 51 times. TCM syndrome, which could reflect the characteristics of TCM, was reported 4 times. 29 outcomes (90.6%, 29/32) were provided definitions or OMIs. Among these outcomes, the outcome measurement times ranged from 0 to 34.

**Conclusions:**

This scoping review provides potential knowledge that should be considered as priority in future research of acupuncture for eczema.

## 1. Introduction

Atopic eczema (AE) is a chronic inflammatory skin disease, clinically characterized by exacerbations and remissions of eczematous skin with inflammation, pruritus and excoriations, scaling, dry skin, and susceptibility for cutaneous bacterial and mycotic infections [1]. In the past 30 years, lifetime prevalence has shown a worldwide increase, which plateaus at 10–20% in developed countries and continues to increase in China and many developing countries but lower [2].

The concurrent use of terms such as “eczema,” “atopic eczema,” “atopic dermatitis,” “atopic eczema/dermatitis syndrome,” and “neurodermatitis” has led to confusion and inconsistency in their application. In China, AE is also called as chronic eczema (CE) [3]. Therefore, the nomenclature review committee of the World Allergy Organization has proposed the use of “eczema” as a unifying term, and this term is also used throughout the text of this review [4].

Acupuncture was frequently applied in cases of allergy [5]. In recent years, it has been increasingly utilized as an adjuvant therapy to conventional treatment of eczema [6]. A large number of clinical trials related to acupuncture for eczema have been published in various journals [7]. However, evidence of acupuncture for eczema keeps unclear. The treatment prescriptions are various, and outcome reporting showing heterogeneity makes it impossible to merge data or compare efficacy of different interventions in a systematic review.

The aim of this scoping review is to identify potential treatment prescriptions and compare the outcome reporting for acupuncture clinical trials with the existing core outcome domains developed by the Harmonizing Outcome Measures for Eczema (HOME) consensus, which may help acupuncturists establish potential prioritized treatment prescriptions and choose appropriate outcomes in their future research.

## 2. Methods

### 2.1. Search Strategy

We conducted a systematic electronic search from Medline, PubMed, Cochrane Library, CBM, CNKI, Wanfang Database, and VIP Database from their inception to September 30, 2020. The search terms included acupuncture, acupoints, electroacupuncture, needle warming therapy, autologous whole blood injection, autohemotherapy, acupoint injection, catgut implantation, filiform steel needle, fire needle, plum-blossom needle, atopic dermatitis, chronic eczema, and atopic eczema. Language was restricted to Chinese and English. The search strategy is shown in the supplementary materials (available here).

### 2.2. Inclusion Criteria and Exclusion Criteria

The inclusion criteria are as follows:

① Population: patients diagnosed with either AD, AE, or CE were included as participants in this study [2, 8, 9]. There were no restrictions on age, sex, race, or region.
② Intervention: the included studies examined the use of acutherapy (acupuncture therapy). The studies could use multimodal interventions, but acutherapy had to be included.
③ Comparison: comparison of the intervention group with the control group in addition to treatment methods other than acutherapy, such as drugs or other forms of traditional Chinese medicine.
④ Outcomes: no restriction.
⑤ Study types: all kinds of clinical studies including randomized clinical trials, observational studies, and case series were included in this scoping review.

The exclusion criteria are as follows:

Comments, animal experiments, and narrative reviews were excluded.

### 2.3. Literature Identification

We imported the results from different databases into NoteExpress 3.2.0 [10] and then deleted duplicate studies. Two reviewers (Zeng Y. J. and Zhang J. L.) conducted data identification independently. They reviewed the abstracts and full texts and extracted relevant information from the included studies. If the studies involved in comparison of therapeutic effects of different acupoint prescriptions, then the most effective acupoint prescription according to the criteria of therapeutic effect was extracted. Any discrepancies were resolved by consensus.

### 2.4. Data Extraction

The following information was extracted pertaining to the reference title, first author's name, publication year, study location, study design, intervention and comparisons, duration, results, and outcome measurements. These data were charted in a custom-made data extraction file and double checked by ZZ.

### 2.5. Data Analysis

To overcome some weaknesses of previous studies and to determine whether acupuncture requires a different outcome set that will reflect particular effects of acupuncture on eczema symptom management, the outcomes reporting was analyzed by description analysis. Two researchers (ZZ and DZ) merged the overlapping outcomes according to the definition of core outcome domains recommended by HOME consensus including signs, symptoms, quality of life, and long-term control and grouped individual outcomes into the appropriate outcome domain independently. In addition, immune system outcomes and adverse events were added to outcome domains. These outcome sets are often preferred in what to consider as evidence relative to efficacy and safety in clinical trials of acupuncture on eczema.

Treatment prescriptions were analyzed by SPSS Modeler 18.0 [11]. We first analyzed the frequency of acupoints and meridians with text mining. Then, we determine common acupoint combinations, the support degree of different acupoint combinations, and the core acupoint association network of acupuncture for eczema by association rule mining. We obtain the core acupoints using hierarchical cluster analysis. We also analyzed the co-occurrence matrix of the top 18 acupoints using Heml 1.0.3.7 [12]. The results are illustrated by a heat map.

## 3. Results

### 3.1. Study Identification

**Figure 1:**
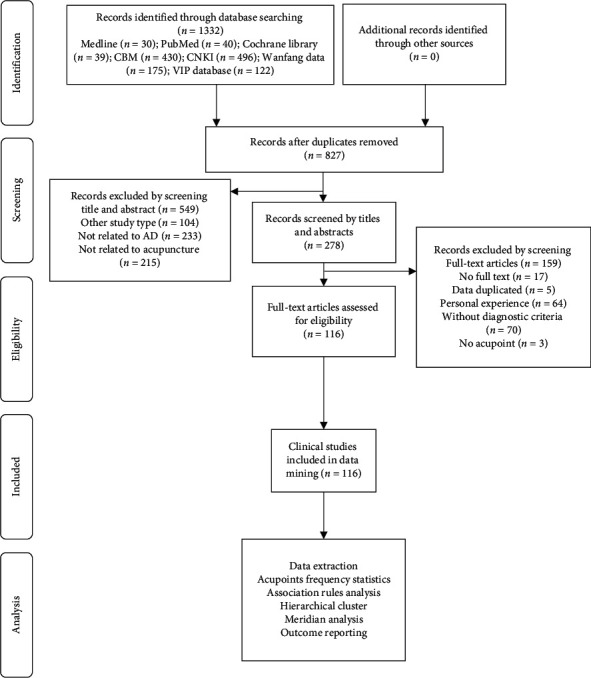
Flow chart of the research process.

A total of 1332 records were identified from the literature search, and a total of 116 eligible studies were included. The flowchart of the review is shown in Figure 1.

### 3.2. Characteristics of the Inclusive Studies

The characteristics of the inclusive studies are shown in Supplementary Table 1. The studies included were published between 1998 and 2020. Most of the studies (81%, 94/116) were published in 2010 and after. The majority of the studies were conducted in China (*n* = 111). The rest of the studies originated from Germany, United States, and Korea. Most of the studies used a randomized controlled trial design with two or three arms (*n* = 91). Nine studies used a control design with simple randomized assignment of participants to the experimental and control groups. 14 studies used a single group design such as a longitudinal prospective design. The duration of treatment sessions ranged from 7 d to 140 d throughout the studies, while two studies did not report duration. The sample sizes of all the studies range from 10 to 323, with an average of 82 participants. Intervention procedures varied among the 116 studies. Most of the studies (44%) included investigated manual acupuncture. Only two studies discussed electroacupuncture. Other studies involved the assessment of acupoint therapies such as autologous whole blood injection (*n* = 21), acupoint injection (*n* = 18), catgut implantation (*n* = 7), fire needle (*n* = 24), and plum-blossom needle (*n* = 7). Non-RCT studies discussed treatments such as acupuncture, autologous whole blood injection, acupoint injection, catgut implantation, fire needle, and plum-blossom needle (*n* = 23). All of the included studies demonstrated statistically significant (*P* < 0.05) improvements in at least one targeted eczema outcome.

### 3.3. Results of Data Mining

#### 3.3.1. Frequency Statistics of Acupoints and Meridians

**Figure 2:**
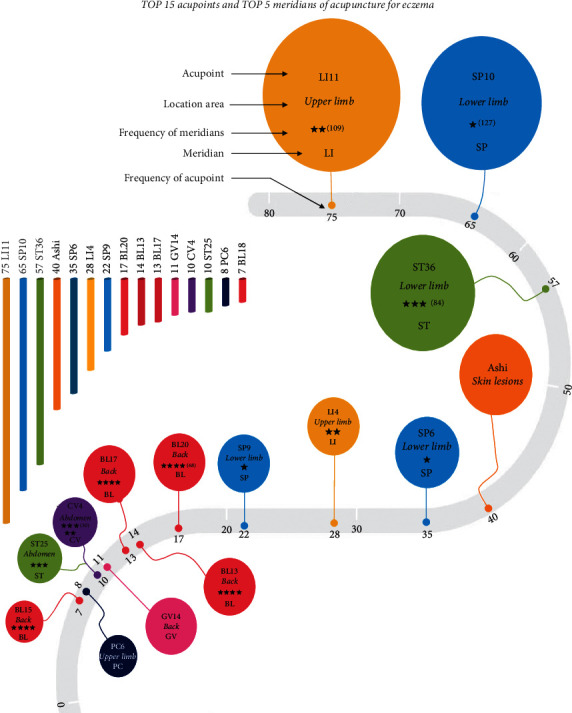
The top 15 acupoints and top 5 meridians used in acupuncture for eczema.

Acupoint frequency was analyzed based on the acquired 116 acupoint prescriptions, which involved 73 acupoints. The top 15 acupoints used frequently were LI11, SP10, ST36, Ashi, SP6, LI4, SP9, BL20, BL13, BL17, GV14, CV4, ST25, PC6, and BL18, in turn. There were 5 acupoints with frequency ≥30, which were Ashi, LI11, SP10, ST36, and SP6. A total of 12 meridians, two extra meridians, and one Ashi point were used in acupuncture for eczema. There are 14 meridians recorded in the 116 acupoint prescription. The top 5 meridians included the Spleen Meridian of Foot-Taiyin (SP), Large Intestine Meridian of Hand-Yangming (LI), Stomach Meridian of Foot-Yangming (ST), Bladder Meridian of Foot-Taiyang (BL), and conception vessel (CV). The most frequently used meridian was SP, which was used 127 times and involved 5 acupoints. Detailed information can be checked in Figure 2.

#### 3.3.2. Common Acupoint Combinations

**Figure 3:**
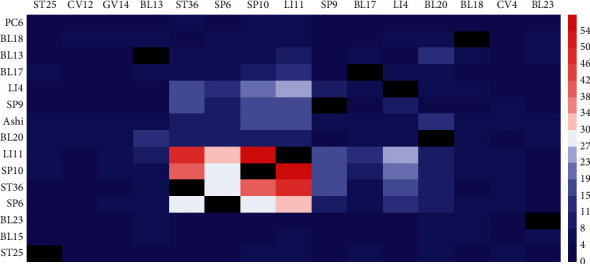
Co-occurrence matrix of acupoints.

A total of 15 common acupoint combinations were frequently used over 17 times. There were four acupoint combinations with frequency ≥35: LI11 and SP10, LI11 and ST36, SP10 and ST36, and LI11, SP10, and ST36. The most frequently used acupoint combination was LI11 and SP10, with 58 times. LI11 and ST36 was used 48 times, SP10 and ST36 was used 40 times, and LI11, SP10, and ST36 was used 37 times. Detailed information can be checked in Table 1. The co-occurrence matrix of the 18 acupoints is presented in Figure 3, which were consistent with the common acupoint combinations.

#### 3.3.3. Association Rules of Acupoints

**Figure 4:**
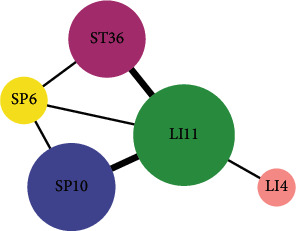
Core acupoint association network of acupuncture for eczema. The size of the circle represents the frequency, and the width of the line indicates the support degree.

There were 13 acupoint combinations with confidence levels ranging more than 80% and support degree more than 20%, of which SP6, SP10⟶LI11 was listed as the association rules of 96.55%, indicating that when SP6, SP10 was selected, the probability of selecting LI11 was 96.55%. Also, the highest support degree was SP10⟶LI11, which was 54.31%. The association rules for acupoints are described in Table 2. The core acupoint association network of acupuncture for eczema is listed in Figure 4. The degree of support from strong to weak was acupoint LI11 combined with SP10, ST36 with LI11, SP6 with LI11, SP6 with SP10, and LI4 with LI11.

### 3.4. Outcomes Reporting in Clinical Trials of Acupuncture for Eczema

For included clinical trials, a total of 6 outcome distinct domains were identified in the 32 outcome measurements (Table 3). Seven (21.9%, 7/32) outcomes were reported only once. The most frequently reported outcome was the eczema area, which was reported 97 times (83.6%, 97/116). Immune system outcomes were assessed in 15 outcome measurements, which totally reported 37 times. Adverse events were reported 51 times. TCM syndrome, which could reflect the characteristics of TCM, was reported 4 times.

29 outcomes (90.6%, 29/32) were provided definitions or OMIs. 17 (53.1%, 17/32) outcomes were provided one OMI or definition, 4 (12.5%, 4/32) outcomes were provided two OMIs or definitions, and 8 (25.0%, 8/32) outcomes were provided more than two OMIs or definitions. In addition, among these outcomes, the outcome measurement times ranged from 0 to 34, and the median time was 6.5. Itch and eczema area had more measurement times than other outcomes did.

## 4. Discussion

The meridians, acupoint combinations, and core acupoints identified from the studies included in this scoping review broadly cover the characteristics of acupuncture prescriptions that could be offered in eczema treatment providing antipruritus and anti-inflammatory effect. Acutherapy offers an area for further clinical practice and research for treating eczema.

From the previous systematic review, we found that the majority of clinical trials of acupuncture for eczema are in low methodological quality and quality of evidence [13–15], so it is difficult to draw a definite conclusion. In addition, the researchers and clinicians may use different acupoints, so it is impossible for some clinical trials to include in systematic review when there is heterogeneity in prescription. From the results of data mining, the core acupoints may provide potential prescription to researchers.

This scoping review suggests that specific outcome measures for future clinical trials of acupuncture are clinician-reported signs, patient-reported symptoms, health-related quality of life and long-term control, TCM syndrome, immune system outcomes, and adverse events, which are different form the HOME. In addition, researchers choose different outcome measurement instruments, so some clinical trials will be excluded from systematic reviews, which produce waste and lower the value of research. For acupuncture clinical trials, following the HOME core outcome set for eczema may help improve the consistency of outcomes and outcome measurement instruments.

The theory of TCM is totally different from that of western medicine. When clinicians use acupuncture, the TCM syndromes are important factors that should be considered in the process of treatment and efficacy evaluation. Therefore, in the core outcome set for eczema, TCM syndromes should be considered. In addition, the HOME is developed by stakeholders from developed countries, and the perspectives of professionals and patients from low- and middle-income countries are missed. Therefore, the core outcome set for eczema should be achieved consensus in researchers, clinicians, and patients in China.

The current research had several strengths. To our knowledge, this was the first study that used bioinformatics methods and searched Chinese and English databases to assess which acupoints have been used to treat eczema. First, previous research was limited in that it could only “qualitatively” interpret the characteristics of acupoint prescriptions used in eczema treatment. We overcame this limitation by extracting significant “quantitative” characteristics using the association rule mining. Second, we explored core acupoints used in eczema treatment. Moreover, we evaluated the quality of outcome reporting, OMIs/definitions, and outcome measurement times.

## 5. Limitations

Our research had several limitations. The present study was based on the frequency of prescription used in the clinical practice and literature. For this reason, we could not evaluate new candidate acupoints emerging from recent experimental studies. Relatedly, we did not evaluate the clinical effectiveness of each prescription and adjunct points prescribed based on syndrome differentiation in our study. Finally, the acupoint combinations and core acupoints of acupuncture for eczema were rarely confirmed by large-scale multicenter clinical trials or animal experiments. Further clinical/experimental studies are needed to assess whether the results derived from our research have real meaning for applications.

## 6. Conclusions

The preference of core acupoints is LI11, SP10, ST36, SP6, and LI4 in acupuncture clinical studies on eczema. In the future, the researchers could focus on them to prove the efficacy of potential core acupoints. However, researchers should use the core outcome set for eczema to improve the consistency of outcomes and outcome measurement instruments so that the clinical trials will be not exclude from systematic reviews because of the heterogeneity of outcome reporting and outcome measurement instruments.

## Figures and Tables

**Table 1:** Common acupoint group of acupuncture for eczema.

No.	Acupoints	Frequency
1	LI11, SP10	58*∗*
2	LI11, ST36	48
3	SP10, ST36	40*∗*
4	LI11, SP10, ST36	37
5	SP6, LI11	31
6	SP6, SP10	29
7	LI11, SP6, SP10	28
8	SP6, ST36	27
9	LI11, SP6, ST36	25
10	LI4, LI11	24
11	ST36, SP6, SP10	23
12	LI4, SP10	20
13	LI4, SP10, LI11	20
14	LI4, ST36	18
15	LI4, ST36, LI11	17

*∗*The frequency of two-acupoint combinations includes that of the two acupoints in three-acupoint combinations.

**Table 2:** Association rules of acupoints.

Number	Acupoints	Confidence level (%)	Support degree (%)
1	SP6, SP10⟶LI11	96.55	25.00
2	SP6⟶LI11	93.94	28.45
3	SP6, ST36⟶LI11	92.59	23.28
4	ST36, SP10⟶LI11	92.50	34.48
5	SP10⟶LI11	92.06	54.31
6	SP6, LI11⟶SP10	90.32	26.72
7	SP6⟶SP10	87.88	28.45
8	ST36⟶LI11	87.27	47.41
9	LI4⟶LI11	85.71	24.14
10	SP6, ST36⟶SP10	85.19	23.28
11	LI4, LI11⟶SP10	83.33	20.69
12	SP6⟶ST36	81.82	28.45
13	SP6, LI11⟶ST36	80.65	26.72

**Table 3:** The outcomes reporting in clinical trials of acupuncture for eczema.

Domains/outcomes	Outcomes reporting (n)	OMIs/definitions	Measurement time point (*n*)
*Clinical signs*			
Erythema	65	SCORAD, EASI, Likert scale, Chinese self-made questionnaire	30
Oozing/crust	21	SCORAD, EASI, Likert scale, POME	18
Induration/edema/papulation	64	SCORAD, EASI, Likert scale, Chinese self-made questionnaire	31
Excoriation	59	SCORAD, EASI, Likert scale, POME, Chinese self-made questionnaire	29
Lichenification	60	SCORAD, EASI, Chinese self-made questionnaire	28
Dryness	9	SCORAD, POME	13
Lesion area	97	SCORAD, EASI, Likert scale, Chinese self-made questionnaire	32
Skin temperature	2	Infrared radiation thermometer	3

*Symptoms*			
Itch	91	VAS, EIQ, POME, Chinese self-made questionnaire	34
Bleeding	1	POME	3
Cracks	1	Chinese self-made questionnaire	2
Sleep loss	10	VAS, ISI, PSQI, SRSS, Likert scale	13
TCM symptoms	4	Chinese self-made questionnaire	8

*Heath-related quality of life*			
Quality of life	20	DLQI, EPQOLS	11

*Long-term control of flares*			
Recurrence rate	20	—	28

*Immune system outcomes*			
BAT	1	FLOW-CAST basophil activation test	3
BASO	1	Blood routine examination	2
EOS	3	Automated hematology analyzer, blood routine examination	5
CD3+	2	Flow cytometry	3
CD4+	4	Flow cytometry	11
CD8+	4	Flow cytometry	11
IFN-*γ*	3	ELISA	4
TNF-*α*	2	ELISA	3
IL-2	3	ELISA	4
IL-4	5	ELISA	5
IL-5	3	ELISA	4
IL-12	1	ELISA	2
IL-18	1	ELISA	2
IgE	5	Immunoturbidimetry assay, ELISA	11
CRP	1	ELISA	3

*Others*			
Adherence/compliance	20	—	0
Adverse events/side effects	51	—	20

BAT: basophil activation test; EOS: eosinophil count; BASO: basophil; CRP: C-reactive protein; EASI: Eczema Area and Severity Index; POEM: Patient-Oriented Eczema Measure; EIQ: Eppendorf Itch Questionnaire; VAS: Visual Analogue Scale; SCORAD: Scoring Atopic Dermatitis Index; DLQI: Dermatology Life Quality Index; EPQOLS: quality of life scale for chronic eczema; ISI: Insomnia Severity Index; PSQI: Pittsburgh Sleep Quality Index; SRSS: Sleep Self-Rating Scale.

## Data Availability

The datasets used and/or analyzed during the current study are available from the corresponding author upon reasonable request.

## Abbreviations

AE: Atopic eczema
AD: Atopic dermatitis
AMED: Japan Agency for Medical Research and Development
ARM: Association rule mining
BASO: Basophil
BAT: Basophil activation test
BL: Bladder Meridian of Foot-Taiyang
CBM: Chinese biomedical medicine
CCT: Clinical control trial
CE: Chronic eczema
CMCC: Chinese medical current content
CNKI: China National Knowledge Infrastructure
CRP: C-reactive protein
DLQI: Dermatology Life Quality Index
EA: Electroacupuncture
EASI: Eczema Area and Severity Index
EIQ: Eppendorf Itch Questionnaire
EOS: Eosinophil count
EPQOLS: Quality of life scale for chronic eczema
ERK: Extracellular regulated protein kinases
GB: Gallbladder Meridian of Foot-Shaoyang
GV: Governor vessel
IFN: Interferon
Ig: Immunoglobulin
IL: Interleukin
ISI: Insomnia Severity Index
LI: Large Intestine Meridian of Hand-Yangming
LR: Liver Meridian of Foot-Jueyin
POEM: Patient-oriented eczema measure
PSQI: Pittsburgh Sleep Quality Index
RCT: Randomized control trial
SCORAD: Scoring Atopic Sermatitis Index
SP: Spleen Meridian of Foot-Taiyin
SRSS: Sleep Self-Rating Scale
ST: Stomach Meridian of Foot-Yangming
TNF: Tissue necrosis factor
VAS: Visual Analogue Scale.

## Supplementary Materials

Supplementary Materials The detailed search strategy and characteristics of the inclusive studies are shown in Supplementary materials. .
