# Supplementary material for: Potential Acupoint Prescriptions and Outcome Reporting for Acupuncture in Atopic Eczema: A Scoping Review
Source: Evid Based Complement Alternat Med. 2021 Jun 26;2021:9994824. doi: 10.1155/2021/9994824 (PMC8257338; doi:10.1155/2021/9994824)
Supplement: Supplementary Materials — The detailed search strategy and characteristics of the inclusive studies are shown in Supplementary materials. . [file 9994824.f1.docx]

**Search strategy**

Pubmed:

#1 ((((randomized controlled trial[Title/Abstract]) OR (controlled trial[Title/Abstract])) OR (clinical trial[Title/Abstract])) OR (case-control studies[Title/Abstract])) OR (case series[Title/Abstract])

#2 ((((((((((((acupuncture[Title/Abstract]) OR (acupoints[Title/Abstract])) OR (body acupuncture[Title/Abstract])) OR (scalp acupuncture[Title/Abstract])) OR (manual acupuncture[Title/Abstract])) OR (Acupoint injection[Title/Abstract])) OR (autologous whole-blood acupoint injection[Title/Abstract])) OR (electroacupuncture[Title/Abstract])) OR (Autohemotherapy[Title/Abstract])) OR (fire needle[Title/Abstract])) OR (plum blossom needle[Title/Abstract])) OR (catgut implantation[Title/Abstract])) OR (filiform steel needle[Title/Abstract])

#3 ((((atopic dermatitis[Title/Abstract]) OR (eczema[Title/Abstract])) OR (chronic eczema[Title/Abstract])) OR (atopic eczema[Title/Abstract]))

#4 #1 and #2 and #3

| **Supplement Table 1.** The characteristics of the inclusive studies | | | | | | | |
| --- | --- | --- | --- | --- | --- | --- | --- |
| **Study** | **Location** | **Design** | **Duration** | **Intervention** | **Comparison** | **Results** | **Outcomes Measures** |
| **Zhou SH. 2016** | **China** | **RCT** | **4 wk, twice a week, 30 min treatments.** | **Acupuncture (four points around navel) (n=29)** | **Acupuncture (n=28)** | **Statistically significant difference in VAS score, DLQI, symptom score (*P＜.05*)** | **VAS, symptom score, DLQI** |
| **F. Pfab et al. 2012** | **USA** | **RCT, double blind** | **Seven-arm crossover trial, different intervention sessions were separated by at least 1 week.** | **Verum acupuncture (both preventive and abortive), placebo acupuncture (both preventive and abortive), verum cetirizine (ingested preventively), placebo cetirizine tablet and a no-intervention control (n=20) (Each patient served as their own control)** | | **A specific effect of acupuncture as well as cetirizine on itch perception and skin reactions compared with placebo and NIs. The timing of acupuncture interventions played a significant role in itch reduction. Abortive acupuncture was superior to these and all other therapy arms.** | **VAS, EIQ, D2 attention test** |
| **Kang SH et al. 2018** | **Korea** | **RCT, double blind** | **4 wk, 3 times/wk, 15 min treatments.4wk observation period.** | **Verum acupuncture 1 (3 times weekly)（n=10), verum acupuncture 2 (twice weekly)(n=10)** | **Sham acupuncture (twice weekly) (n=10)** | **The SCORAD, VAS (Pruritus), VAS (Insomnia), POEM, DLQI, and EASI were significantly improved in the VA groups. There was no significant difference between VA1 and VA2 groups in all the main evaluation indices.** | **SCORAD, VAS, POEM, DLQI, EASI** |
| **Quan XH et al. 2014** | **China** | **RCT** | **12 wk, 3 times/wk.** | **Flying needle combinated with oral medicine (n=29)** | **Oral medicine (n=30)** | **Statistically significant difference in SCORAD (P＜.05) after treat 12 weeks** | **SCORAD** |
| **F. Pfab et al. 2011** | **Germany** | **RCT, single blind** | **33 d, twice a week, 30 min treatments.** | **Acupuncture (n=5)** | **No treatments (n=5)** | **Statistically significant difference in VAS score, basophil activation (P＜.05), no statistically significant difference in SCORAD.** | **SCORAD, VAS, BAT** |
| **F. Pfab et al. 2009** | **Germany** | **RCT, double blind** | **11 min treatments.** | **Verum-points acupuncture (VA) (n=10)** | **Placebo-points acupuncture (PA)(n=10), no acupuncture (NA)(n=10)** | **Statistically significant difference in VAS score, EQI (P<.001), wheal and flare sizes, perfusion (P＜.05)** | **VAS, LASER-Doppler, EIQ** |
| **Lee HC. 2019** | **Korea** | **RCT, single blind** | **4 wk, 3 times/wk, 20 min treatments.** | **Acupuncture (n=7)** | **Needle-embedding (n=7)** | **The SCORAD index, TEWL, skin hydration and DLQI were significantly improved in both groups (P<.05), but no significant difference between the 2 groups.** | **SCORAD, TEWL, Skin-O-Mat, DLQIY** |
| **Meng XH et al. 2015** | **China** | **RCT** | **4 wk, 30 min/d** | **Electroacupuncture combined with topical medication (n=12)** | **Topical medication (n=10), electroacupuncture (n=10)** | **Significantly reduced symptom score (P<0.05)** | **SSRI** |
| **Zhao DP et al. 2007** | **China** | **CCT** | **7 d, once a day, 30-40 min treatments.** | **Acupuncture combined with autohemotherapy (n=100)** | **Medication (n=90)** | **Statistically significant difference in symptom score (P＜0.01)** | **-** |
| **Liu HH. 2015** | **China** | **RCT** | **45 d, once every 4 d, 12 sessions** | **Autoblood acupoint injection (n=47)** | **Autoblood intramuscular injection (n=47)** | **Statistically significant difference in symptom score (P＜.05)** | **-** |
| **Chen F et al. 2015** | **China** | **Case series** | **-** | **Catgut implantation (n=25)** | | **Clinically significant improvement.** | **-** |
| **Wu YF. 2020** | **China** | **RCT** | **14 d, once a day, 20 min treatments.** | **Acupuncture combined with traditional chinese medicine (n=30)** | **Traditional chinese medicine (n=30)** | **Statistically significant difference in EASI index, DLQI, itching score (P＜.05)** | **EASI, DLQI** |
| **Wang YM. 2019** | **China** | **RCT** | **6 wk, once every 3 wk, 4 wk observation period.** | **Acupoint injection (n=64)** | **Topical medication (n=64)** | **Statistically significant difference in EASI index, itching score (P ＜ .05)** | **EASI** |
| **Wu SY. 2018** | **China** | **RCT** | **14 d, once a day, 30 min treatments.** | **High-frequency electroacupuncture (n=20), low-frequency electroacupuncture (n=20)** | **Medication (n=20)** | **The SSS, Skindex-16 score, IL-12 were significantly improved in both groups (P<.05), the curative effect of HF group was better.** | **SSS, Skindex-16, ELISA** |
| **Liu Y. 2013** | **China** | **RCT** | **20 d, once every 2 d, 10 sessions, 30 min treatments.** | **Acupuncture (n=32)** | **Fire needle (n=33)** | **Statistically significant difference in EASI index (P ＜ .05)** | **EASI** |
| **Xiao GF et al. 2000** | **China** | **Case series** | **3m, once every 7-10 d** | **Autohemotherapy (n=216)** | | **Clinically significant improvement.** | **-** |
| **Liu K. 2017** | **China** | **RCT** | **8 kw, 3 times/wk, 30 min treatments.** | **Acupuncture combined with fire needle (n=30)** | **Topical medication (n=30)** | **Statistically significant difference in EASI index (P ＜ .05) after treat 6 wk. Significantly reduced VAS score and EPQOLS score in both groups (P < .01), but no significant difference between the 2 groups.** | **EASI, VAS, EPQOLS** |
| **Song T. 2017** | **China** | **RCT** | **5 wk, twice a week, 10 sessions.** | **Fire needle combined with traditional Chinese medicine (n=30)** | **Traditional Chinese medicine (n=31)** | **The EASI index was significantly improved in both groups (P<.05), but no significant difference between the 2 groups. Statistically significant difference in itching score (P ＜ .05)** | **EASI** |
| **Jiao ZH et al. 2015** | **China** | **RCT** | **12 wk, once a week, 12 sessions.** | **Fire needle combined with acupuncture (n=28)** | **Fire needle (n=29), acupuncture (n=29), medicine (n=29)** | **Significant difference with the medicine group (P < .05)** | **EASI** |
| **Li ST. 2017** | **China** | **Case series** | **8 wk, twice a week, 16 sessions.** | **Fire needle combined with acupuncture (n=47)** | | **Clinically significant improvement (P < .05).** | **VAS, DLQI, HAMA, HAMD, Infrared thermography** |
| **Zhou ZH et al. 2020** | **China** | **RCT** | **8 wk, once a week.** | **Fire needle combined with topical medication (n=50)** | **Topical medication (n=50)** | **Significant difference with the control group (P < .05)** | **-** |
| **Song L. 2018** | **China** | **RCT** | **3 wk, once every 2 d.** | **Fire needle combined with three oxygen blood acupoint injection (n=30)** | **Topical medication (n=30)** | **Statistically significant difference in VAS score, EASI index (P＜.05)** | **EASI, VAS** |
| **Gao H et al. 2015** | **China** | **CCT** | **12 wk, once a week, 12 sessions.** | **Fire needle combined with acupuncture (n=28)** | **Fire needle (n=29), acupuncture (n=29), medicine (n=29)** | **Significant difference with the medicine group (P < .05)** | **VAS, EASI** |
| **Gu Z. 2017** | **China** | **RCT** | **4 wk, once every 2 d** | **Fire needle combined with traditional Chinese medicine (n=35)** | **Traditional chinese medicine (n=33), oral medicine (n=33)** | **Statistically significant difference in EASI index, itching score (P ＜ .05)** | **EASI** |
| **Gulxan Turdi. 2020** | **China** | **RCT** | **20 d, once every 4 d.** | **Fire needle combined with traditional uyghur medicine (n=39)** | **Traditional uyghur medicine (n=39)** | **Statistically significant difference in EASI index, DLQI, itching score (P＜.05), but no significant difference in serum EOS, BASO** | **EASI, DLQI, ELISA** |
| **Liang FL. 2016** | **China** | **RCT** | **3 wk, 3 times/wk.** | **Fire needle (n=30)** | **Topical medication (n=30)** | **Statistically significant difference in EASI index (P＜.05)** | **EASI** |
| **Yang K et al. 2020** | **China** | **RCT** | **Fire needle: 4 wk, 2 times/wk, 10-15 min treatments. Autohemotherapy: 4 wk, once a week, 5 min treatments.** | **Fire needle combined with autohemotherapy (n=30)** | **Acupuncture (n=30)** | **Statistically significant difference in EASI index, VAS score (P ＜ .05)** | **EASI, VAS** |
| **Hou JY et al. 2014** | **China** | **Case series** | **-** | **Fire needle (n=30)** | | **Clinically significant improvement.** | **-** |
| **Wang XQ et al. 2015** | **China** | **CCT** | **4 wk, once a week.** | **Fire needle (n=30)** | **Topical medication (n=30)** | **Statistically significant difference in EASI index, itching score (P ＜ .05)** | **EASI** |
| **Wang GX. 2011** | **China** | **RCT, single blind** | **3 wk, once every 5 d.** | **Fire needle (n=32)** | **Topical medication (n=30)** | **Statistically significant difference in EASI index, itching score (P ＜ .01)** | **EASI** |
| **Huang S et al. 2004** | **China** | **RCT** | **3 wk, once every 3 d.** | **Fire needle (n=35)** | **Topical medication (n=32)** | **Significant difference with the control group (P < .01)** | **-** |
| **Jia HL et al. 2016** | **China** | **RCT** | **4 wk, twice a week.** | **Fire needle (n=30)** | **Topical medication (n=30)** | **Statistically significant difference in EASI index, VAS score (P ＜ .05)** | **EASI, VAS** |
| **Liu Y et al. 2018** | **China** | **RCT** | **1 m, twice a week.** | **Fire needle (n=33)** | **Oral medicine (n=33)** | **Statistically significant difference in EASI index, itching score, the serum IL-18, IgE level, EOS (P ＜ .05)** | **EASI, ELISA, Flow cytometry** |
| **Zhou PH. 2016** | **China** | **RCT** | **21 d, once every 4 d, 6 sessions.** | **Fire needle (n=32)** | **Topical medication (n=31)** | **Statistically significant difference in EASI index (P ＜ .01)** | **EASI** |
| **Wang R. 2019** | **China** | **RCT** | **2m, once every 14 d, 3 sessions.** | **Catgut implantation combined with traditional Chinese medicine (n=33)** | **Catgut implantation combined with oral medicine (n=32), traditional Chinese medicine (n=34)** | **Statistically significant difference in EASI, itching score, DLQI, serum IgE level (P ＜ .05)** | **EASI, DLQI, ELISA** |
| **Wei JH et al. 2017** | **China** | **RCT** | **21 d, once a day, 30 min treatments.** | **Acupuncture combined with traditional Chinese medicine (n=60)** | **Traditional Chinese medicine (n=60)** | **Statistically significant difference in symptom score, serum IL-4, IL-5 level and CD4+, CD8+ (P ＜ .05)** | **ELISA, flow cytometry** |
| **Yun JX. 2019** | **China** | **RCT** | **4 wk, once every 2 d, 30 min treatments.** | **Acupuncture (n=35)** | **Acupuncture (different acupoints) (n=35)** | **Statistically significant difference in itching score, EASI index (P ＜ .05)** | **EASI** |
| **He CB et al. 2011** | **China** | **RCT** | **48 d, once every 4 d, 12 sessions, 5 min treatments.** | **Autoblood acupoint injection (n=60)** | **Autoblood intramuscular injection (n=60)** | **Statistically significant difference in symptom severity (P ＜ .05)** | **-** |
| **Tang J. 2016** | **China** | **RCT** | **4 wk, 3 times/wk, 30 min treatments.** | **Acupuncture 1 (n=29)** | **Acupuncture 2 (n=30)** | **Statistically significant difference in EASI index, VAS score, EPQOLS (P ＜ .05)** | **EASI, VAS, EPQOLS** |
| **Liu Y et al. 1999** | **China** | **CCT** | **once every other day, 5-15 sessions.** | **Acupoint injection (n=40)** | **Medication (n=31)** | **Statistically significant difference in symptom severity (P ＜ .01)** | **-** |
| **Chen TJ. 2010** | **China** | **RCT** | **2 m, once every 3 d, 5 min treatments.** | **Acupoint injection combined with autohemotherapy (n=66)** | **Medication (n=52)** | **Statistically significant difference in EASI index (P ＜ .05)** | **EASI** |
| **Li YS. 2018** | **China** | **RCT** | **4 wk, 3 times/wk.** | **Fire needle (n=30)** | **Acupuncture (n=30), medication (n=30)** | **Statistically significant difference in EASI index, VAS score, DLQI score (P ＜ .01)** | **EASI, VAS, DLQI** |
| **Li Q et al. 2018** | **China** | **RCT** | **4 wk, 3 times/wk.** | **Fire needle (n=30)** | **Oral medicine (n=30)** | **Statistically significant difference in EASI index, VAS score, DLQI score, the serum IFN-γ, IL-4 level (P ＜ .05).** | **EASI, VAS, DLQI, ELISA** |
| **Zhang YH et al. 2011** | **China** | **RCT** | **4 wk, once every 3 d.** | **Plum-blossom needle (n=48)** | **Topical medication (n=47)** | **Significant difference with the control group (P < .05)** | **-** |
| **Lin WQ et al. 2008** | **China** | **RCT** | **3 m, once every 2 wk, 5 sessions.** | **Plum-blossom needle (n=31)** | **Acupoint injection (n=40)** | **Significant difference with the control group (P < .05)** | **-** |
| **Yu DR. 2006** | **China** | **RCT** | **28 d, once every 4 d.** | **Plum-blossom needle combined with acupoint injection (n=58)** | **Oral medicine (n=58)** | **Statistically significant difference in CD3+, CD4+, CD8+, and the serum IL-2, IL-4 level (P ＜ .01)** | **ELISA, flow cytometry** |
| **Wang J et al. 2009** | **China** | **RCT** | **2 wk, once every other day** | **Plum-blossom needle combined with topical medication (n=33)** | **Topical medication (n=32)** | **Significant difference with the control group (P < .05)** | **-** |
| **Zhou YY. 2017** | **China** | **RCT** | **10 d, once a day.** | **Plum-blossom needle combined with medication (n=30)** | **Medication (n=30)** | **Significant difference with the control group (P < .05)** | **SRSS, EPQOLS** |
| **Zhu H et al. 2010** | **China** | **Case series** | **3 m, once a week, 10 sessions.** | **Plum-blossom needle (n=40)** | | **Clinically significant improvement.** | **-** |
| **Yang WJ et al. 2011** | **China** | **RCT** | **40 d, once every 20 d.** | **Acupoint injection combined with soya bean oil (n=34)** | **Soya bean oil (n=31), topical medicine (n=30)** | **Statistically significant difference in symptom severity (P ＜ .01)** | **-** |
| **Yang YX. 2009** | **China** | **RCT** | **4 wk, once every 10-14 d, 5 min treatments.** | **Acupoint injection (n=50)** | **Topical medication (n=50)** | **Statistically significant difference in symptom severity (P ＜ .01)** | **-** |
| **Tang JM. 2020** | **China** | **RCT** | **2-4 wk, once a week.** | **Acupoint injection (n=64)** | **Medication (n=48)** | **Significant difference with the control group (P < .001)** | **-** |
| **Wu B. 2008** | **China** | **RCT** | **45 d, once a week.** | **Acupoint injection combined with medicine (n=106)** | **Medication (n=102)** | **Significant difference with the control group (P < .05)** | **-** |
| **Han LC. 2007** | **China** | **RCT** | **46 d, once a week.** | **Acupoint injection combined with medicine (n=86)** | **Medication (n=83)** | **Significant difference with the control group (P < .05)** | **-** |
| **Gao YJ et al. 2018** | **China** | **RCT** | **3 wk, once a week.** | **Acupoint injection (LI4) (n=42)** | **Acupoint injection (Ashi point) (n=38)** | **No significant difference** | **-** |
| **Lv JJ. 2010** | **China** | **CCT** | **30 d, 2-3 times/wk.** | **Autohemotherapy combined with medicine (n=60)** | **Medication (n=60)** | **Significant difference with the control group (P < .05)** | **-** |
| **Zhang L et al. 2017** | **China** | **RCT** | **3 wk, 5 times/wk.** | **Three oxygen autoblood acupoint injection (n=59)** | **Autoblood (n=59), NaCl (n=59) acupoint injection** | **Statistically significant difference in EASI index, itching score (P ＜ .05).** | **EASI** |
| **Wang YH et al. 2020** | **China** | **RCT** | **8 wk, once a week.** | **Autohemotherapy and fire needle combined with medicine (n=35)** | **Medication (n=35)** | **The serum IgE level, CRP level, VAS, DLQI were significantly reduced in both groups (P < .05), the treatment group was better (P < .05).** | **ELISA, VAS, DLQI** |
| **Xie XL et al. 2011** | **China** | **RCT** | **8 wk, once every 3 d.** | **Autohemotherapy combined with medicine (n=68)** | **Medication (n=43)** | **Significant difference with the control group (P < .05)** | **-** |
| **Peng WT. 2013** | **China** | **RCT** | **4 wk, once every 3 d.** | **Autohemotherapy (n=28)** | **Traditional Chinese medication (n=30)** | **The EASI index, itching score, DLQI were significantly improved in both groups (P < .01), and significant difference between the 2 groups (P < .05).** | **EASI, DLQI** |
| **Zhan HJ. 2015** | **China** | **RCT** | **6 wk, once a week.** | **Acupoint injection (n=30)** | **Traditional Chinese medication (n=30)** | **Significant difference with the control group (P < .05)** | **-** |
| **Fu F. 2018** | **China** | **RCT** | **-** | **Fire needle combined with traditional Chinese medicine (n=30)** | **Traditional Chinese medication (n=30)** | **Significant difference with the control group (P < .05)** | **EASI** |
| **Tang J et al.2019** | **China** | **RCT** | **4 wk, 3 times/wk, 30 min treatments.** | **Acupuncture 1 (n=29)** | **Acupuncture 2 (n=30)** | **EASI index was significantly improved in both groups (P < .05), but no significant difference between the 2 groups. Statistically significant difference in VAS score (P ＜ .05)** | **ELISA, VAS** |
| **Feng YM et al. 2019** | **China** | **RCT** | **1 wk, once a day.** | **Acupuncture (n=100)** | **Medication (n=100)** | **Significant difference with the control group (P < .05)** | **-** |
| **Peng Y et al. 2015** | **China** | **CCT** | **30 d, once every 3 d.** | **Acupuncture (n=50)** | **Medication (n=50)** | **Significant difference with the control group (P < .06)** | **-** |
| **Ma J et al. 2017** | **China** | **CCT** | **2 wk, 1-2 times/d.** | **Acupuncture (n=35)** | **Medication (n=35)** | **Significant difference with the control group (P < .07)** | **-** |
| **Tan Y et al. 2019** | **China** | **RCT** | **1 m, 5 times/m.** | **Catgut implantation (n=45)** | **Oral medicine (n=45)** | **Statistically significant difference in serum IFN-γ, TNF-α, IL-4, IL-2, IL-5 level(P ＜ .05)** | **ELISA** |
| **Li Y et al. 2018** | **China** | **RCT** | **60 d, once every 15 d.** | **Catgut implantation combined with traditional Chinese medicine (n=28)** | **Traditional Chinese medication (n=29)** | **Statistically significant difference in EASI index (P ＜ .05)** | **EASI** |
| **Yang XS et al. 2019** | **China** | **RCT** | **2 m, once a week.** | **Catgut implantation combined with traditional Chinese medicine (n=31)** | **Traditional Chinese medication (n=31)** | **Statistically significant difference in EASI index, itching score (P ＜ .05)** | **EASI** |
| **Li K et al. 2018** | **China** | **RCT** | **35 d, once a week.** | **Catgut implantation (n=42)** | **Oral medicine (n=42)** | **Serum IFN-γ, TNF-α, IL-4, IL-2, IL-5 level were significantly induced in both groups, and significant difference between the 2 groups (P < .05).** | **ELISA** |
| **Bian YF et al. 2008** | **China** | **RCT** | **2 wk, 3 times/wk, 5 sessions.** | **Acupoint injection (n=38)** | **Medication (n=28)** | **Significant difference with the control group (P < .05)** | **-** |
| **Liang DM et al. 2012** | **China** | **RCT** | **4 wk, 1 time per 2 weeks.** | **Acupoint injection combined with medicine (n=48)** | **Medication (n=48)** | **Significant difference with the control group (P < .01)** | **-** |
| **Li Y. 2010** | **China** | **RCT** | **1 wk, once every 2 d.** | **Acupoint injection combined with medicine (n=32)** | **Medication (n=46)** | **Significant difference with the control group (P < .01)** | **-** |
| **Wei JX et al. 2002** | **China** | **Case series** | **15 d, once every 3 d.** | **Acupoint injection (n=323)** | | **Clinically significant improvement.** | **-** |
| **Zhao SY et al. 2005** | **China** | **RCT** | **2-3 m, once a week.** | **Acupoint injection combined with medicine (n=32)** | **Medication (n=46)** | **Significant difference with the control group (P < .01)** | **-** |
| **Hu YM et al. 2014** | **China** | **RCT** | **30 d, once a week.** | **Acupoint injection combined with medicine (n=39)** | **Medication (n=39)** | **Significant difference with the control group (P < .05)** | **-** |
| **Xu YP et al. 2004** | **China** | **RCT** | **3 wk, once a week.** | **Acupoint injection combined with medicine (n=51)** | **Im. combined with medicine (n=26), medicine (n=20)** | **Significant difference with the control group (P < .05)** | **-** |
| **Chen K. 2004** | **China** | **RCT** | **20 d, once a day.** | **Acupoint injection (n=30)** | **Oral medicine (n=35)** | **Significant difference with the control group (P < .01)** | **-** |
| **Deng YL et al. 2016** | **China** | **RCT** | **120-140 d, once every 6-7 d.** | **Autohemotherapy combined with plum-blossom needle (n=40)** | **Autohemotherapy (n=20), plum-blossom needle(n=20)** | **Significant difference with two control group (P < .05)** | **-** |
| **Feng H et al. 2020** | **China** | **Case series** | **5 wk, once a week.** | **Autohemotherapy (n=28)** | | **Clinically significant improvement.** | **EASI** |
| **Liu ZM. 2013** | **China** | **RCT** | **4 wk, twice a week.** | **Autohemotherapy combined with medicine (n=20)** | **Medication (n=20)** | **Significant difference with the control group (P < .05)** | **-** |
| **Zhou YF. 2018** | **China** | **CCT** | **4 wk, twice a week, 30 min treatments.** | **Fire needle combined with acupuncture (n=30)** | **Medication (n=30)** | **The EASI index, VAS score, DLQI were significantly improved in both groups (P<.05), but EASI index and DLQI no significant difference between the 2 groups. And statistically significant difference in VAS score (P ＜ .05)** | **EASI, VAS, DLQI** |
| **Xie CC et al. 2011** | **China** | **RCT** | **4 wk, 3 times/wk.** | **Acupuncture combined with medicine (n=20)** | **Acupuncture (n=20), medicine (n=20)** | **Significant difference with two control group (P < .05)** | **-** |
| **Wang YM et al. 2013** | **China** | **RCT** | **2 wk, 3 times/wk, 30 min treatments.** | **Acupuncture (n=44)** | **Topical medication (n=42)** | **Statistically significant difference in EASI index, VAS score (P ＜ .01)** | **EASI, VAS** |
| **Cheng SR et al.2014** | **China** | **RCT** | **12 wk, 3 times/wk, 30 min treatments.** | **Acupuncture combined with traditional Chinese medicine (n=29)** | **Traditional Chinese medicine (n=30)** | **Statistically significant difference in SCORAD index, VAS score, DLQI score, ISI score (P ＜ .05)** | **SCORAD, VAS, DLQI, ISI** |
| **Xie CC et al. 2009** | **China** | **RCT** | **4 wk, 3 times/wk, 30 min treatments.** | **Acupuncture combined with medicine (n=20)** | **Medication (n=18)** | **Significant difference with the control group (P < .05)** | **-** |
| **Deng JJ. 2018** | **China** | **RCT** | **2 wk, acupuncture: once a day, 30 min treatments; autohemotherapy: once every 2 d.** | **Acupuncture combined with autohemotherapy (n=20)** | **Medication (n=20)** | **Statistically significant difference in EASI index, VAS score, DLQI score (P ＜ .05)** | **EASI, VAS, DLQI** |
| **Yi JC. 2012** | **China** | **RCT** | **20 d, once a day, 30 min treatments.** | **Acupuncture combined with topical medication (n=48)** | **Oral medicine combined with topical medication (n=32)** | **Significant difference with the control group (P < .05)** | **SSRI** |
| **Cai X et al. 2011** | **China** | **RCT** | **10 d, once a day, 30 min treatments.** | **Acupuncture combined with medication (n=34)** | **Medication (n=28)** | **Significant difference with the control group (P < .05)** | **-** |
| **Wei Y. 2009** | **China** | **RCT** | **4 wk, once a day, 30 min treatments.** | **Acupuncture combined with traditional Chinese medicine (n=21)** | **Traditional Chinese medicine (n=20)** | **Significant difference with the control group (P < .05) after 2 wk treating, but no significant difference after 4 wk treating.** | **-** |
| **Mai GW. 2014** | **China** | **RCT** | **3 wk, 3 times/wk, 30 min treatments.** | **Acupuncture combined with traditional Chinese medicine (n=32)** | **Traditional Chinese medicine (n=30)** | **Statistically significant difference in EASI index, VAS score, EPQILS score (P ＜ .05)** | **EASI, EPQILS, VAS** |
| **Fan JH. 2016** | **China** | **RCT** | **4 wk, once every other day, 20 min treatments.** | **Acupuncture combined with traditional Chinese medicine (n=30)** | **Traditional Chinese medicine (n=31)** | **Statistically significant difference in EASI index, itching score (P ＜ .05)** | **EASI** |
| **Yan JX. 2018** | **China** | **RCT** | **60 d, once every other day, 30 min treatments.** | **Acupuncture (n=25)** | **Medication (n=25)** | **Significant difference with the control group (P < .05)** | **-** |
| **Xu HX. 2018** | **China** | **RCT** | **40 d, once every other day.** | **Acupuncture combined with oral medicine (n=30)** | **Topical medicine combined with oral medication (n=30)** | **Statistically significant difference in EASI index, VAS score (P ＜ .05)** | **EASI, VAS** |
| **Chang H. 2013** | **China** | **Case series** | **3-8 wk, twice per week** | **Acupuncture (n=50)** | | **Clinically significant improvement.** | **-** |
| **Cui JX. 2009** | **China** | **Case series** | **Twice per week, 5 sessions.** | **Acupuncture (n=68)** | | **Clinically significant improvement.** | **-** |
| **Wang XY et al. 2006** | **China** | **Case series** | **1-10 wk, 30 min treatments.** | **Acupuncture (n=65)** | | **Clinically significant improvement.** | **-** |
| **Ding L et al. 2000** | **China** | **Case series** | **1-10 wk, 30 min treatments.** | **Acupuncture (n=57)** | | **Clinically significant improvement.** | **-** |
| **Gong JZ. 2015** | **China** | **RCT** | **1 wk, 1-2 times/d.** | **Acupuncture (n=27)** | **Topical medicine (n=32)** | **Significant difference with the control group (P < .01)** | **-** |
| **Li M et al. 2011** | **China** | **RCT** | **3 wk, once every 3 d.** | **Acupoint injection combined with plum-blossom needle (n=36)** | **Medication (n=34)** | **Significant difference with the control group (P < .05)** | **-** |
| **Zhang ZP. 2010** | **China** | **RCT** | **1 wk, once a day, 30 min treatments.** | **Acupuncture (n=54)** | **Topical medicine (n=54)** | **Significant difference with the control group (P < .01)** | **-** |
| **Xu HY et al. 2013** | **China** | **RCT** | **20 d, once a day.** | **Acupuncture (n=42)** | **Medication (n=38)** | **Statistically significant difference in EASI index (P ＜ .01)** | **EASI** |
| **Wang LJ. 2016** | **China** | **Case series** | **20 d, once a day.** | **Acupuncture (n=88)** | | **Statistically significant difference in EASI index (P ＜ .05)** | **EASI** |
| **Yuan XL et al. 1998** | **China** | **Case series** | **20 d, once a day.** | **Acupuncture (n=55)** | | **Clinically significant improvement.** | **-** |
| **Lv SL. 2016** | **China** | **Case series** | **20-30 d, once a day, 20 min treatments.** | **Acupuncture (n=49)** | | **Clinically significant improvement.** | **-** |
| **Jiang ZX. 2016** | **China** | **RCT** | **12 wk, 3 times/wk, 30 min treatments.** | **Acupuncture combined with traditional Chinese medicine (n=30)** | **Traditional Chinese medicine (n=29)** | **Statistically significant difference in SCORAD index, DLQI score, itching score (P ＜ .05)** | **SCORAD, DLQI** |
| **Liu J. 2016** | **China** | **RCT** | **12 wk, 3 times/wk, 30 min treatments.** | **Acupuncture combined with traditional Chinese medicine (n=27)** | **Traditional Chinese medicine (n=27)** | **Statistically significant difference in SCORAD index, PSQI index, VAS score, SRSS score (P ＜ .05)** | **SCORAD, PSQI, VAS, SRSS** |
| **Cao FF. 2016** | **China** | **RCT** | **4 wk, twice per week, 30 min treatments.** | **Acupuncture combined with traditional Chinese medicine (n=32)** | **Traditional Chinese medicine (n=30)** | **Statistically significant difference in EASI index, itching score, serum IgE level (P ＜ .05)** | **EASI, ELISA** |
| **Wu YX. 2011** | **China** | **RCT** | **39 d, once every other day, 30 min treatments.** | **Acupuncture combined with traditional Chinese medicine (n=41)** | **Traditional Chinese medicine (n=35)** | **The EASI index, DLQI score, itching score were significantly induced in both groups (P < .01), and significant difference between the 2 groups (P < .01).** | **EASI, DLQI** |
| **Chen XH et al.2016** | **China** | **RCT** | **12 wk, 3 times/wk, 30 min treatments.** | **Acupuncture combined with medicine (n=30)** | **Acupuncture (n=29), medicine (n=31)** | **Statistically significant difference in SCORAD index, PSQI index, VAS score, SRSS score (P ＜ .05)** | **SCORAD, PSQI, VAS, SRSS** |
| **Zhou KW et al. 2019** | **China** | **RCT** | **10 d, once every day.** | **Fire needle combined with traditional Chinese medicine (n=60)** | **Traditional Chinese medicine (n=60), topical medicine (n=60)** | **Significant difference with the control group (P < .05)** | **-** |
| **Wei YY. 2017** | **China** | **RCT** | **12 wk, once every 5 d.** | **Autohemotherapy combined with nursing care (n=60)** | **Medicine combined with nursing care (n=60), medicine (n=60)** | **Significant difference with the control group (P < .05)** | **-** |
| **Wu ZQ et al.2013** | **China** | **RCT** | **4 wk, once every other day.** | **Autohemotherapy combined with medicine (n=61)** | **Medicine (n=46), autohemotherapy (n=58)** | **Significant difference with the control group (P < .05)** | **.** |
| **Xu BY.2019** | **China** | **RCT** | **2 wk, twice per week.** | **Autohemotherapy (n=35)** | **Medicine (n=35)** | **Statistically significant difference in DLQI score (P ＜ .05), but no significant difference in EASI index, VAS score.** | **EASI, DLQI, VAS** |
| **Zuo Z et al. 2010** | **China** | **CCT** | **30 d, twice per week.** | **Autohemotherapy (n=10)** | **Medicine (n=10)** | **Significant difference with the control group (P < .05)** | **-** |
| **Chen YL et al.2013** | **China** | **RCT** | **60 d, once every 3 d.** | **Autohemotherapy (n=100)** | **Medicine (n=100)** | **The CD3+, CD4+, CD8+ were significantly induced in both groups (P < .05), but no significant difference.** | **Flow cytometry** |
| **EASI: Eczema Area and Severity Index; POEM: Patient Oriented Eczema Measure; EIQ: Qualitative assessment of itch intensity; VAS: visual analogue scale; SCORAD: Scoring atopic dermatitis index; SSS: Simple scoring systems of eczema; TEWL: Trans Epidermal Water Loss; STAXI: State-Trait Anger Expression Inventory; DLQI: Dermatology Life Quality Index; EPQOLS: Quality of life Scale for chronic eczema; ISI: Insomnia severity index; PSQI: Pittsburgh sleep quality index; SRSS: Sleep self-rating scale** | | | | | | | |
